# Sterols from the Madagascar Sponge *Fascaplysinopsis* sp

**DOI:** 10.3390/md8122961

**Published:** 2010-12-17

**Authors:** Maurice Aknin, Emmanuelle Gros, Jean Vacelet, Yoel Kashman, Anne Gauvin-Bialecki

**Affiliations:** 1 Laboratoire de Chimie des Substances Naturelles et des Sciences des Aliments, Faculté des Sciences et Technologies, Université de la Réunion, 15 Avenue René Cassin, BP 7151, 97715 St Denis Messag Cedex 9, La Réunion, France; E-Mails: maurice.aknin@univ-reunion.fr (M.A.); emagros@orange.fr (E.G.); 2 Centre d’Océanologie de Marseille, Aix-Marseille Université, CNRS UMR 6540 DIMAR, Station Marine d’Endoume, rue Batterie des Lions, 13007 Marseille, France; E-Mail: jean.vacelet@univmed.fr (J.V.); 3 School of Chemistry Tel Aviv University, Sackler Faculty of Medicine, Tel Aviv University, Ramat Aviv 69978, Israel; E-Mail: kashman@post.tau.ac.il (Y.K.)

**Keywords:** *Fascaplysinopsis* sp., Dictyoceratida, Δ^5,7^-sterols, sponge-associated microorganisms, chemotaxonomy

## Abstract

The sponge *Fascaplysinopsis* sp. (order Dictyoceratida, Family Thorectidae) from the west coast of Madagascar (Indian Ocean) is a particularly rich source of bioactive nitrogenous macrolides. The previous studies on this organism led to the suggestion that the latter should originate from associated microsymbionts. In order to evaluate the influence of microsymbionts on lipid content, 10 samples of *Fascaplysinopsis* sp. were investigated for their sterol composition. Contrary to the secondary metabolites, the sterol patterns established were qualitatively and quantitatively stable: 14 sterols with different unsaturated nuclei, Δ^5^, Δ^7^ and Δ^5,7^, were identified; the last ones being the main sterols of the investigated sponges. The chemotaxonomic significance of these results for the order Dictyoceratida is also discussed in the context of the literature. The conjugated diene system in Δ^5,7^ sterols is known to be unstable and easily photo-oxidized during storage and/or experiments to produce 5α,8α-epidioxy sterols. However, in this study, no 5α,8α-epidioxysterols (or only trace amounts) were observed. Thus, it was supposed that photo-oxidation was avoided thanks to the natural antioxidants detected in *Fascaplysinopsis* sp. by both the DPPH and β-caroten bleaching assays.

## 1. Introduction

In continuation of our long-standing interest in the chemistry of marine sponges, we have investigated the Madagascar *Fascaplysinopsis* sp. sponge (order Dictyoceratida, family Thorectidae). Previous studies on different batches of this sponge, resulted in the isolation and identification of four unprecedented groups of cytotoxic sponge nitrogenous macrolides, *i.e.*, salarins A–J [1–3], tulearins A–C [1,4], taumycins A and B [5], and a fourth group, combining taumycin and salarin, designated tausalarin C [6]. All four groups are novel classes of marine natural compounds with rare unprecedented functional moieties. The structural similarity of the various sponge metabolites of the four groups to microorganism and fungal metabolites (e.g., the cyanobacteria *Lyngbia bouillonii* metabolites madangolide and laingolide A) [7,8] suggested that these compounds originate from guest microorganisms rather than from the host sponge itself. This notion is supported by the chemical content variations from one collection to the other; *Fascaplysinopsis* sp. was indeed collected on two occasions from the west coast of Madagascar in Salary Bay in January 2007 and in February 2008. Moreover, sponges are well known to be hosts for a large community of microorganisms, which comprise a significant percentage (up to 50–60%) of the biomass of the sponge host [9,10]. Based on bacterial community studies employing molecular methods such as Denaturing Gradient Gel Electrophoresis (DGGE), 16S rRNA gene sequencing and Fluorescence *In Situ* Hybridization (FISH), it has been recognized that the sponge-associated bacterial community consists of several bacterial phyla such as Proteobacteria, Nitrospira, Cyanobacteria, Bacteriodetes, Actinobacteria, Chloroflexi, Planctomycetes, Acidobacteria, Poribacteria and Verrucomicrobia besides members of the domain Archea [10–12]. Other symbiotic microbial populations that inhabit sponges are fungi and microalgae [10–12]. Bacterial symbionts are acquired by a sponge according to two pathways: (1) selective absorption of specific bacteria from the large diversity of bacteria in the surrounding water column that passes through the sponge during filter feeding; (2) vertical transmission of symbionts through the gametes of the sponge by inclusion of the bacteria in the oocytes or larvae [13]. The role of these microbes in sponge biology varies from source of nutrition to mutualistic symbiosis. Symbiotic functions that have been attributed to microbial associates include, in particular, bioactive secondary metabolites production [14,15] and sponges of the orders Halichondrida, Poecilosclerida and Dictyoceratida are known to be a rich source of compounds produced by associated microorganisms [13].

On the basis of the above mentioned literature data and our experimental results, exclusively devoted to the secondary metabolites of *Fascaplysinopsis* sp., we decided to pursue our study by drawing our attention to the lipids of the sponge. Lipids have proved to be also biomarkers for symbiotic microorganisms [16,17]. The purpose of our work was to find out if, like the secondary metabolites, a variation in lipids content will also be observed between specimens. To elucidate this question, 10 specimens of *Fascaplysinopsis* sp were collected from different locations all over the distribution area of this species in Salary Bay, and their lipids investigated. Herein, we report the isolation of the free sterol fraction from the 10 studied specimens and the investigation of the sterol content by high-performance liquid chromatography coupled to ultraviolet diode array detection (HPLC-UV), gas chromatography coupled with flame ionization detection (GC-FID) and gas chromatography coupled to mass spectrometry (GC-MS) methods to elucidate their chemical composition. A suggestion concerning the use of these sterols as markers for the position of *Fascaplysinopsis* sp. within the order Dictyoceratida, is also given.

## 2. Results and Discussion

**Figure 1:**
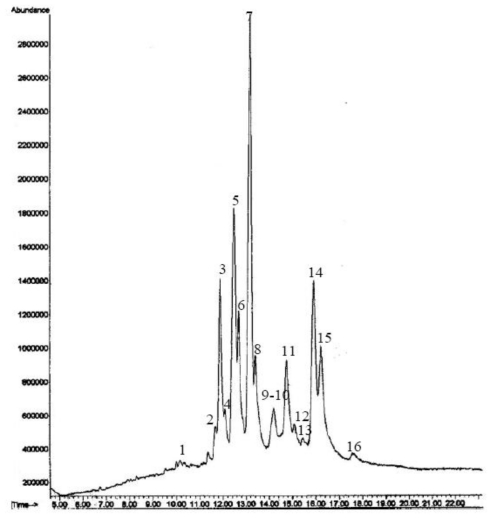
GC-MS chromatogram of the free sterols extracted from *Fascaplysinopsis* sp.

The sterol profiles obtained by GC-MS and GC-FID were exactly identical for all 10 studied specimens of *Fascaplysinopsis* sp. Hence, Figure 1 and Table 1 give results only for one specimen. Figure 1 shows the GC-MS profile of the free sterols. Table 1 reports qualitative and quantitative data obtained, respectively, from GC-MS and GC-FID analyses. The components are listed in order of their elution (relative retention time, RRT) on the SPB-5 column. A classification based on the side chain length and the degree of unsaturation in the nucleus is also summarized in Table 2. The chromatographic analyses allowed the detection and quantification of 16 sterols, among which 14, accounting for 82.9% of the total sterols, were identified. The sterol fraction of *Fascaplysinopsis* sp. mainly consists of:

- 7.1% of conventional mono-unsaturated Δ^5^-sterols including cholesta-5,24-dien-3β-ol (or desmosterol). The latter constitutes near half of the Δ^5^-sterols (3.2%).
- 18.1% of conventional mono-unsaturated Δ^7^-sterols represented by cholest-7-en-3β-ol (10.5%) and 24-methylcholesta-7,22*E*-dien-3β-ol (7.6%).
- 61.0% of known di-unsaturated nucleus Δ^5,7^-sterols with a preponderance of 24-methylcholesta-5,7,22*E*-trien-3β-ol (19.5%), cholesta-5,7,22*E*-trien-3β-ol (12.2%), cholesta-5,7-dien-3β-ol (9.9%) and 24-ethylcholesta-5,7-dien-3β-ol (8.4%).

**Figure 2:**
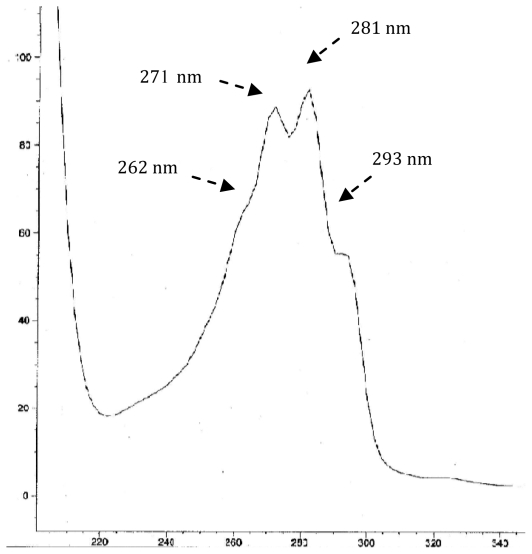
Typical UV absorption for Δ^5,7^-sterols.

**Figure 3:**
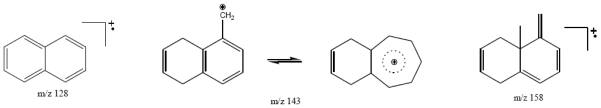
Ions reported to be characteristic in the mass spectra of Δ^5,7^-sterols [19]

Regarding the distribution of nuclei types (Table 2), the Δ^5,7^ sterols (61.0%) appeared to be predominant (Δ^5^:Δ^7^:Δ^5,7^ 1:2.5:8.5). The presence of these conjugated dienes were characterized, on one hand, by their absorption peaks at 262, 271, 281 and 293 nm [18] observed in their UV spectra obtained by HPLC-UV (Figure 2), and on the other hand, by their characteristic fragment ions m/z 128, 143, 157, 158 and 159 [19] observed in their mass spectra obtained by GC-MS (Figure 3). Δ^5,7^ sterols are an interesting group of sterols found in different marine invertebrates. However, this class of sterols is a relatively poorly investigated group of marine sterols, one of the reasons being their instability causing difficulties at two levels, extraction and analyses.

**Figure 4:**
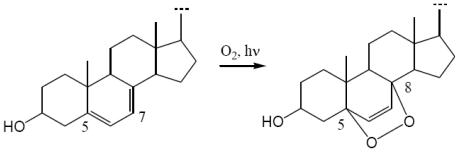
Photooxidation reaction of Δ^5,7^-sterols: formation of 5α,8α-epidioxysterols.

Concerning first “Δ^5,7^ sterols *extraction*”, it is very common for Δ^5,7^-sterols to be accompanied by a high proportion of 5α,8α-epidioxy sterols. The conjugated diene system in Δ^5,7^-sterols can easily be photooxidized during storage and/or chromatographic separation [20–22] (Figure 4). To avoid Δ^5,7^-sterols photooxidation, Arreguin-Espinosa *et al.* [21] proposed two methods for the investigation of marine invertebrates rich in di-unsaturated Δ^5,7^ sterols: (1) the fresh marine invertebrate must be dipped in alcohol and transported as soon as possible to the laboratory, where it must be extracted immediately in the dark; or (2) marine invertebrates should be stored in the dark with 0.1% antioxidant such as BHA (Butylated HydroxyAnisole). For the 10 studied specimens of *Fascaplysinopsis*, no specific precaution was taken and yet none of the potential artifact 5α,8α-epidioxy sterol (or only trace amounts) was detected. At the same time, a sponge *Liosina paradoxa* was investigated by our research group using the same experimental procedure and its main steroidal constituents appeared to be Δ^5^ sterols and 5α,8α-epidioxy sterols. Besides, while in *Fasacplysinopsis* sp., natural antioxidants were detected by both the DPPH and β-carotene bleaching assays (Table 3), in *Liosina paradoxa* no antioxidant compound was revealed. On the basis of these results, we can suggest that the natural antioxidants detected in *Fascaplysinopsis* sp. are able to prevent the fast photooxidation of native Δ^5,7^-sterols.

**Figure 5:**
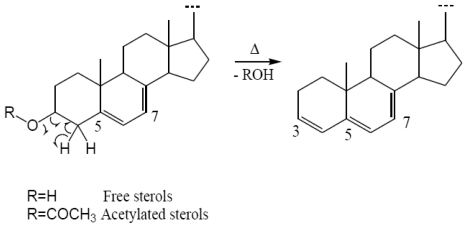
Dehydration (deacetylation) of Δ^5,7^ sterols (free and acetylated).

**Figure 6:**
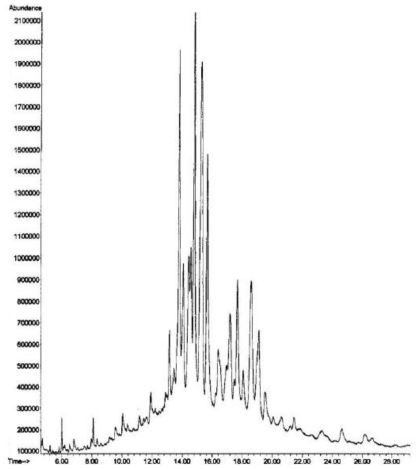
GC-MS chromatogram of the acetylated sterols: multiplications of peaks.

Concerning “Δ^5,7^ sterols *analyses*” it is worth noting that the free or acetylated Δ^5,7^ sterols are very thermally labile and their dehydration (deacetylation) can be observed under GC conditions when raising the temperature of the injector and the column (Figure 5). This process is indeed thermodynamically more favorable for Δ^5,7^ sterols than for mono-unsaturated Δ^5^ or Δ^7^ sterols due to the formation of a longer conjugated system after the formation of the C-3 double bond. Thus, on the GC chromatogram, the resulting Δ^3,5,7^ trienes can be observed in mixture with the remaining sterols (free or esterified) [19,23]. With the experimental conditions used for *Fascaplysinopsis* sp., no dehydration occurred for the free sterols, contrary to the steryl acetates for which strong deacetylation was observed leading to multiplication and overlapping peaks on GC chromatograms (Figure 6). Quantitative determination was therefore established from the free sterols chromatograms.

Sponges are an important source of compounds that may be employed as phylogenetic characters to aid in unraveling the numerous sponge classification problems [24]. Difficulties encountered in sponge classification are clearly due to their simple organization resulting in a lack of clear morphological markers required for a robust phylogenetic reconstruction. Today, molecular biology and chemical composition have been both used in complement to morphological characters. Concerning more precisely the chemical composition, the use of secondary metabolites as chemotaxonomic tools is more and more common because these compounds increase in number from year to year and offer a structural complexity promising a large source of new characters. Presence (or absence) of a particular compound or compound family among different sponge taxa may indicate a closer phylogenetic relationship. However, high abundance of microsymbionts in sponges, which may be the source of some of the secondary metabolites, bears severe difficulties for chemotaxonomy. The assignment of a compound as sponge or symbiont compound is indeed difficult, and in a general manner, symbiont compounds cannot be used as taxon markers as long as the symbionts may switch between hosts—the genealogic information would therefore be lost. Successful applications of symbiont compounds in chemotaxonomy are dependent on the stability of the host-symbiont relationship. Some symbionts might show high host specificity and their compounds could therefore be considered as suitable markers. This could be the case for symbionts acquired by vertical transmission through the gametes of the sponge. Manifestly, for the sponge *Fascaplysinopsis* sp., the variation observed in secondary metabolites content, probably due to a variation of microbial communities in the sponge, prevents us to use these compounds as solid chemotaxonomic markers. On the other hand, the stability of sterol content (qualitatively and quantitatively) evidenced in this study, leads us to suppose that sterols are a more suitable chemotaxonomic tool than secondary metabolites in the case of *Fascaplysinopsis* sp.

Like most of the sponges of the order Dictyoceratida (Table 4), *Fascaplysinopsis* sp. contains predominantly Δ^5,7^-sterols. Biosynthetic experiments showed that in marine invertebrates, Δ^5,7^ sterols are intermediates in the transformation of dietary Δ^5^-sterols to Δ^7^-sterols [20,33,34]. This transformation of sterols could be the result of a so-called biochemical coordination. Sterols coming from the diet are transformed in order to satisfy the membrane requirements of the sponge. Indeed, in the animal kingdom, cholesterol is known to play a functional role in the cell membrane. However, marine invertebrates such as sponges often contain sterols with modified skeletal or with additional alkyl groups in the side chain, instead of cholesterol, in their cell membrane [35]. Thus, it was suggested by Santalova *et al*. [36] that the peculiarities of free sterol fractions in marine animals could be connected to their own toxic activities. Introduction of unusual sterols could make membranes less sensitive to the actions of the species own membranolytic toxins.

Concerning more precisely the transformation of Δ^5^-sterols to Δ^7^-sterols through Δ^5,7^-sterols, many unsolved questions remain. For example, questions remain regarding the 7-dehydrogenase responsible for the transformation of Δ^5^-sterols to Δ^5,7^-sterols. This transformation was proved to occur rapidly because it generates a thermodynamically more stable conjugated system. However, we do not know if there are some sterols preferred by 7-dehydrogenase. John *et al.* [34] founded that in *Ciocalypta* sp. (Order Halichondrida, Family Halichondriidae), the sterols with 24-alkyl groups were greatly preferred by the enzyme. In contrast, in *Dysidea fragilis*, *Dysidea herbacea* and *Dysidea avara* (Order Dictyoceratida, Family Dysideidae), sterols containing side-chain double bonds, especially those with C-22 double bonds, were preferred by the 7-dehydrogenase [20]. At last, De Rosa *et al.* [32] showed that in *Fasciospongia cavernosa* (Order Dictyoceratida, Family Thorectidae), sterols with unsaturated side chains, as well as 24-alkylated sterols, were preferred by the 7-dehydrogenase. From Table 5, it is evident that in *Fascaplysinopsis* sp. (Order Dictyoceratida, Family Thorectidae) like *Fasciospongia cavernosa*, sterols with C-22 unsaturated side chains (40.2%), as well as 24-alkylated sterols (37.2%) are preferred by the 7-dehydrogenase. Among the Dictyoceratida sponges, the structural preferences of 7-dehydrogenase enable us to distinguish the Dysideidae sponges (Dysidea spp.) from the Thorectidae sponges (*Fasciospongia* and *Fascaplysinopsis* spp.). More investigations are clearly needed on different Dictyoceratida species in order to clarify the taxonomic value of, first of all, the presence of Δ^5,7^-sterols in Dictyoceratida, and secondly, the structural preferences of 7-dehydrogenase within the different families of this order.

## 3. Experimental Section

### 3.1. Sponge material

Ten specimens (SP01-SP10) of *Fascaplysinopsis* sp. were collected in October 2009 at a depth of 25–30 m from 6 sites spread throughout the known distribution area of the sponge (Salary Bay *ca.* 100 km north of Tuléar, Madagascar). Each sample was labeled, frozen immediately and kept at −20 °C until processed; its location was recorded using a Global Positioning System (GPS) receiver (Table 6). Voucher specimens (# MHNM.16119.0) were deposited in the Museum d’Histoire Naturelle de Marseille, Palais Longchamp, 1 Bd Philippon, 13004 Marseille, France.

### 3.2. Extraction and isolation of sterols

Each of the 10 specimens of *Fascaplysinopsis* sp. was homogenized in a Waring-Blender and extracted with CHCl_3_/MeOH (2:1 v/v) at room temperature. The mixture was filtered, and the organic phase was concentrated *in vacuo* to yield the crude extract, which was subjected to middle pressure liquid chromatography on silica gel eluted with: (1) isohexane/EtOAc (95:5 v/v), (2) EtOAc and (3) EtOAc/MeOH (80:20 v/v) to afford three fractions F1, F2 and F3 respectively. The EtOAc fractions (F2) were then chromatographed on a silica gel column, using increasing concentration of EtOAc in iso-hexane. From the fractions eluted with isohexane/EtOAc (95:5 to 90:10 v/v), free sterols were obtained. They were further purified by methanol recrystallization. The yields of the recovered free sterols estimated on the basis of the weight of the crude extracts are reported in Table 6. A part of the free sterols was acetylated overnight at room temperature using a mixture of acetic anhydride/pyridine (1:1 v/v).

### 3.3. Analysis of sterols

Sterols were analyzed in free form by GC-FID (quantitative analysis) and GC-MS (qualitative analysis):

GC analyses were carried out using a Varian Gas chromatograph Model CP-3800 equipped with a flame ionization detection (FID) system and a non polar SPB-5 capillary column (60 m × 0.32 mm I.D., film thickness 0.25 μm). The oven temperature was programmed from 260 °C to 300 °C at 4 °C/min and then held isothermally at 300 °C for 30 min. Injector and detector temperatures were maintained at 260 °C and 300 °C, respectively. Nitrogen was used as the carrier gas at a flow rate of 1.0 mL/min. Samples were injected in the splitless mode (injection volume, 0.5 μL of sterols diluted in CH_2_Cl_2_).

GC-MS analyses were conducted on a Hewlett-Packard 6890 series-5972 GC-MS system equipped with a SPB-5 column (60 m × 0.32 mm i.d., film thickness 0.25 μm). The oven temperature was programmed from 260 °C to 300 °C at a rate of 5 °C/min, held for 30 min. The injector and the transfer line were both programmed to 260 °C. Mass spectra were recorded at 30–550 m/z using an ionization energy of 70 eV and a ion source temperature of 200 °C. Helium was used as the carrier gas at a flow rate of 1.1 mL/min. All analyses were done using a 20:1 split ratio (injection volume, 0.5 μL of sterols diluted in CH_2_Cl_2_).

HPLC-DAD analyses were run on an Agilent CPL/SM 1100 series (Massy, France) equipped with LC/MSD Chemstation software, degasser G1322A, binary pump G1312A, autosampler G1313A, thermostated column oven G1316A, diode array detection system G1315B to monitor at all wavelengths from 200 to 400 nm. A LiChrospher 100 RP-18 column (250 × 4.6 mm i.d., 5 μm, Merck, Darmstadt, Germany), joined with a guard column LichroCART 4-4 (Merck) was used. No column thermostat was applied. Separations of the free sterols were carried out using a flow rate of 1 mL/min with isocratic elution of the mobile phase MeOH:H_2_O (95:5 v/v). The injection volume of the sterols diluted in CH_2_Cl_2_ was 10 μL.

### 3.4. Free radical scavenging activity

**Figure 7:**
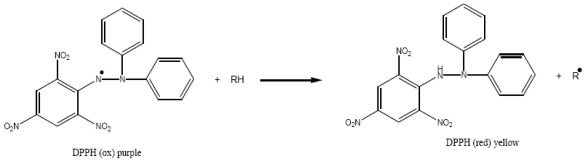
DPPH reduced by a donor of hydrogen.

The chemical test used to evaluate the potential free radical scavenging activity of *Fascaplysinopsis* sp. is based on the method of Takao *et al.* [36]. The detection of this activity is founded on the principle of capture of free radicals provided by 2,2-diphenyl-1-picrylhydrazyl (DPPH). When DPPH is reduced by a donor of hydrogen, its original purple color turns to yellow-pale (Figure 7).

Three fractions (F1, F2, F3) obtained from the crude extract MPLC separation were spotted on two aluminum-backed TLC plates (Silica gel 60 GF_254_) and developed in two solvent systems: isohexane/EtOAc (80:20 v/v) for the first TLC plate and isohexane/EtOAc (20:80 v/v) for the second one. After developing and drying, TLC plates were sprayed with a DPPH solution in MeOH (2 mg.mL^-1^) and examined after 10 min. Compounds with capacity to reduce DPPH appear as yellow spots against a purple background.

### 3.5. Antioxidant activity

A rapid evaluation of antioxidant activity of *Fascaplysinopsis* sp. was determined by the β-caroten bleaching method [38]. The three fractions (F1, F2, F3) obtained from the crude extract MPLC separation were spotted on two aluminum-backed TLC plates (Silica gel 60 GF_254_) and developed in two solvent systems: isohexane/EtOAc (80:20 v/v) for the first TLC plate and isohexane/EtOAc (20:80 v/v) for the second one. After developing and drying, TLC plates were sprayed with a β-carotene solution in CH_2_Cl_2_ (0.5 mg.mL^−1^). Plates were placed under natural light until discoloration of background. The yellow spots remaining indicated the presence of antioxidants.

## 4. Conclusions

From the above discussion, it can be concluded that contrary to the secondary metabolites, the sterols of the Madagascar *Fascaplysinopsis* sp. sponge have a stable composition marked with the dominant presence of Δ^5,7^ diunsaturated sterols. Thus, in this particular case, sterols are not influenced by the variation of bacteria-sponge association that, however, seems to occur in *Fascaplysinopsis* sp. Like many other sponges [39], it was then demonstrated that *Fascaplysinopsis* sp. has both a qualitative and quantitative sterol fingerprint. In consequence, thanks to this stability, the sterol pattern may be useful for taxonomic purposes and it has been suggested that the presence of Δ^5,7^ sterols, *i.e*., the ability to transform the dietary Δ^5^ sterols into Δ^5,7^ sterols through the action of 7-dehydrogenase, may constitute one character shared by members of the order Dictyoceratida. However, much effort should be directed to the 7-dehydrogenase and its specific action which may be used to distinguish two families within the order Dictyoceratida: the Dysideidae for which C-22 double bonds are preferred by the 7-dehydrogenase, and the Thorectidae for which C-22 unsaturated side chains and 24-alkylated sterols are equally submitted to the action of the 7-dehydrogenase.

## Figures and Tables

**Table 1:** Sterol composition (%) of *Fascaplysinopsis* sp.

	Sterols				
		
	Name	Molecular Mass	Short Désignation	RRT^a^	Composition (%)
1	24-Norcholesta-5,7,22*E*-trien-3β-ol	368	C26 Δ^5,7,22^	0.84	1.7
2	Cholesta-5,22*E*-dien-3β-ol	384	C27 Δ^5,22^	0.94	1.5
3	Cholesta-5,7,22*E*-trien-3β-ol	382	C27 Δ^5,7,22^	0.98	12.2
4	Cholesta-5,24-dien-3β-ol	384	C27 Δ^5,24^	1.00	3.2
5	Cholesta-5,7-dien-3β-ol	384	C27 Δ^5,7^	1.04	9.9
6	Cholest-7-en-3β-ol	386	C27 Δ^7^	1.05	10.5
7	24ξ-Methylcholesta-5,7,22*E*-trien-3β-ol	396	C28 Δ^5,7,22^	1.09	19.5
8	24ξ-Methylcholesta-7,22*E*-dien-3β-ol	398	C28 Δ^7,22^	1.12	7.6
9	Unknown^c^	396	C28	1.16	1.7
10	24ξ-Methylcholesta-5,7-dien-3β-ol	398	C28 Δ^5,7^	1.18	1.4
11	24ξ-Ethylcholesta-5,7,22-trien-3β-ol	410	C29 Δ^5,7,22^	1.22	3.5
12	24ξ-Ethylcholest-5-en-3β-ol	414	C29 Δ^5^	1.25	2.4
13	24ξ-Ethylcholesta-5,7,24(24’)-trien-3β-ol	410	C29Δ^5,7,24(24’)^	1.28	1.1
14	24ξ-Ethylcholesta-5,7-dien-3β-ol	412	C29 Δ^5,7^	1.33	8.4
15	Unknown^d^	410	C29 Δ^5,7,22^	1.35	3.3
16	24ξ-Methylcholesta-7-en-3β-ol	414	C29 Δ^7^	1.35	tr b

a RRT (relative retention time) are given for free sterols. RRT of cholesterol = 1.00

b *tr*: trace (% < 0.1%)

c: m/z : 396 (M^+^, 57); 363 (100); 337(36); 255(20); 251 (40); 143(41,5); 60 (39,5); 55 (36)

d: m/z : 410 (M^+^, 76); 377 (100); 351(21,5); 271 (13), 211(23,5); 159(26); 143(46), 55(51,5)

**Table 2:** Sterol composition of *Fascaplysinopsis* sp. according to the side chain length and the degree of unsaturation in the nucleus.

		Side chain length				
		C_26_	C_27_	C_28_	C_29_	Total
**Unsaturation in the nucleus**	**Δ^5^**		4.7		2.4	**7.1**
	**Δ^7^**		10.5	7.6	*tr*	**18.1**
	**Δ^5,7^**	1.7	22.1	20.9	16.3	**61.0**
	**Total**	**1.7**	**37.3**	**28.5**	**18.7**	**86.2**

**Table 3:** Preliminary evaluation of the free radical scavenging activity and the antioxidant capacity of *Fascaplysinopsis* sp. by 2,2-diphenyl-1-picrylhydrazyl (DPPH) and β-carotene bleaching assays.

	F1	F2	F3
**DPPH test**	−	+	−
β**-carotene test**	−	+	−

F1, F2, F3: fractions obtained from the crude extract separation by MPLC.

− absence of active compounds;

+ presence of active compounds.

**Table 4:** Sterol composition of Dictyoceratida sponges according to the degree of unsaturation in the nucleus.

Sponges		Unsaturation in the nucleus				[Ref]
		Δ^0^	Δ^5^	Δ^7^	Δ^5,7^	
Order Dictyoceratida						
	Family Spongidae					
	*Coscinoderma* sp.		+			[25]
	*Hippospongia* sp.		+	+		[25]
	*Ircinia campana*				+	[22]
	*Ircinia foetida*		+	+	+	[26]
	*Ircinia muscarum*		+		+	[27,28]
	*Ircinia pipetta*		+		+	[29]
	*Ircinia* sp.		+		+	[25]
	*Ircinia spinosula*				+	[27]
	*Ircinia variabilis*		+	+	+	[26]
	*Hyattella intestinalis*		+	+		[25]
	*Spongia nitens*				+	[27]
	*Spongia officinalis*				+	[27]
	Family Dysideidae					
	*Dysidea avara*		+		+	[25,27]
	*Dysidea fragilis*	+	+		+	[20]
	*Dysidea herbacea*	+	+	+	+	[30]
	*Dysidea* sp.		+		+	[25]
	*Dysidea tupha*		+			[31]
	Family Thorectidae					
	*Cacospongia* sp.		+	+	+	[25]
	*Fascaplysinopsis* sp.		+		+	[25]
	*Fasciospongia carvernosa*		+	+	+	[32]
	*Luffariella* sp.		+	+	+	[25]
	*Psammocinia* sp.		+	+	+	[25]
	*Thorectandra excavatus*		+		+	[25]
	*Thorecta* sp.		+		+	[25]

+ presence detected.

**Table 5:** Δ^5,7^-sterols from *Fascaplysinopsis* sp. according to side-chain unsaturation and 24-alkylation.

Side chains		without C-24 alkylation		with C-24 alkylation		total

Saturated	Δ^5,7^	-	9.2	1.4	8.4	19.7

unsaturated	Δ^5,7,22^	1.7	12.2	19.5	6.8	40.2
	Δ^5,7,24^	-	-	-	1.1	1.1

total		1.7	22.1	20.9	16.3	61.0

**Table 6:** Localization (GPS coordinates) of the 10 studied *Fascaplysinopsis* specimens and percentage yields of the recovered free sterols.

Zone	GPS coordinates	Specimens	Yield (%)
1	22 32.958S–43°13.070E	**01**	6.0%
		**02**	4.4%
2	22°31.513S–43°12.904E	**03**	5.2%
3	22°30.954S–43°12.636E	**04**	6.1%
		**05**	5.5%
4	22°30.952S–43°12.558E	**06**	3.1%
5	22°30.766S–43°12.635E	**07**	5.6%
		**08**	3.8%
6	22°31.822S–43°12.939E	**09**	4.0%
		**10**	5.2%
